# La vejez y la nueva CIE-11: posición de la Academia Latinoamericana de Medicina del Adulto Mayor

**DOI:** 10.26633/RPSP.2021.112

**Published:** 2021-08-16

**Authors:** Carlos Cano-Gutierrez, Luis Miguel Gutiérrez-Robledo, Roberto Lourenço, Pedro Paulo Marín, Fernando Morales Martínez, José Parodi, Leocadio Rodríguez Mañas, Clemente Humberto Zúñiga Gil

**Affiliations:** 1 Pontificia Universidad Javeriana Bogotá Colombia Pontificia Universidad Javeriana, Bogotá, Colombia.; 2 Instituto Nacional de Geriatría Ciudad de México México Instituto Nacional de Geriatría, Ciudad de México, México.; 3 Pontificia Universidad Católica de Río de Janeiro Río de Janeiro Brasil Pontificia Universidad Católica de Río de Janeiro, Río de Janeiro, Brasil.; 4 Pontificia Universidad Católica de Chile Santiago Chile Pontificia Universidad Católica de Chile, Santiago, Chile.; 5 Universidad de Costa Rica San José Costa Rica Universidad de Costa Rica, San José, Costa Rica.; 6 Universidad San Martín de Porres Lima Perú Universidad San Martín de Porres, Lima, Perú.; 7 Hospital Universitario de Getafe Madrid España Hospital Universitario de Getafe, Madrid, España.; 8 Hospital General de Tijuana Tijuana México Hospital General de Tijuana, Tijuana, México.

**Keywords:** Clasificación Internacional de Enfermedades, envejecimiento, envejecimiento saludable, International Classification of Diseases, aging, healthy aging, Classificação Internacional de Doenças, envelhecimento, envelhecimento saudável

## Abstract

Desde 1948, la Organización Mundial de la Salud ha venido publicando un sistema codificado de causas de enfermedad y muerte bajo el nombre genérico de Clasificación Estadística Internacional de Enfermedades (CIE), con revisiones en profundidad cada 10-15 años. En su última versión, CIE-11, se utiliza una terminología para caracterizar la vejez como “períodos geriátricos inicial y final”, lo que implica una medicalización de esta etapa de la vida que ha generado confusión y polémica. En este trabajo se discute la nueva terminología propuesta a la luz del conocimiento actual en torno a la vejez y el proceso de envejecimiento, y su definición más aceptada. La CIE no solo clasifica las enfermedades sino también los períodos de la vida y los “problemas relacionados con la salud”, y la vejez por sí sola no representa un problema relacionado con la salud para muchos de quienes se encuentran en esta etapa de la vida. Desde esta perspectiva, es imprescindible cambiar o matizar el epígrafe “vejez” de la CIE-11 para que no se perciba como síntoma, signo o resultado clínico anómalo, e introducir términos que reflejen mucho mejor el estado de envejecimiento patológico. Entre los términos que gozan de un creciente soporte experimental y bibliográfico están “fragilidad” y “pérdida de la capacidad intrínseca”, que aportan mucha mayor precisión a la hora de definir la condición de la persona que no goza de un envejecimiento saludable.

## Abstract (en)

Since 1948, the World Health Organization has been publishing the *International Statistical Classification of Diseases and Related Health Problems* (ICD), a coded system of causes of disease and death with an in-depth revision every 10-15 years. In its latest revision, the ICD-11 uses nomenclature characterizing old age as “initial and final geriatric periods,” implying the medicalization of this stage of life, which has created confusion and sparked controversy. This article discusses the new nomenclature proposed, given the current knowledge about old age and the aging process and its most accepted definition. The ICD not only classifies diseases but periods of life and “health-related problems,” and old age *per se* is not a health-related problem for many people at this stage of life. It is therefore essential to change or provide a more nuanced definition of the term “old age” in the ICD-11, so it is not perceived as a symptom, sign, or anomalous clinical outcome, and to introduce nomenclature that more accurately reflects pathological aging. Two terms that are enjoying growing experimental and bibliographic support are “fragility” and “loss of intrinsic capacity,” which offer greater precision when defining the condition of a person who is not experiencing healthy aging.

## RESUMO

Desde 1948, a Organização Mundial da Saúde publica um sistema codificado de causas de doença e morte sob o nome de Classificação Estatística Internacional de Doenças e Problemas Relacionados à Saúde (CID), com revisões profundas a cada 10-15 anos. Sua última versão, a CID-11, utiliza determinada terminologia para caracterizar a velhice como “períodos geriátricos iniciais e finais”, o que implica uma medicalização dessa fase da vida que tem gerado confusão e polêmica. Este artigo discute a nova terminologia proposta à luz dos conhecimentos atuais sobre a velhice e o processo de envelhecimento, e sua definição mais aceita. A CID não classifica apenas as doenças, mas também períodos da vida e “problemas de saúde”, sendo que a velhice por si só não representa um problema de saúde para muitos que se encontram nesta fase da vida. A partir dessa perspectiva, é imprescindível alterar ou qualificar o verbete “velhice” da CID-11 para que não seja percebido como sintoma, sinal ou resultado clínico anormal, e em seu lugar introduzir termos que melhor reflitam o estado de envelhecimento patológico. Entre os termos que têm crescente suporte experimental e bibliográfico estão “fragilidade” e “perda de capacidade intrínseca”, que proporcionam uma precisão muito maior na definição da condição de quem não goza de um envelhecimento saudável.

Desde hace más de un siglo, y con el fin de aportar información relevante a los sistemas de salud nacionales para organizarse en función de las necesidades de la población, se vienen registrando las causas que describen, explican o se asocian con el fallecimiento de las personas. A partir de esos registros se calcula la mortalidad, uno de los principales indicadores de mala salud y cuya reducción es uno de los objetivos principales de los sistemas de salud. Desde 1893, el Instituto Internacional de Estadísticas comenzó a registrar los fallecimientos según las causas de muerte, un proceso adoptado y dirigido desde su sexta edición, en 1948, por la Organización Mundial de la Salud (OMS). Desde entonces se publica bajo el nombre genérico de Clasificación Internacional de Enfermedades (CIE), un sistema codificado de causas de enfermedad y muerte que se actualiza cada 2-3 años, con revisiones en profundidad cada 10-15 años (1).

La versión actual, la CIE-10, está vigente desde 1992 y será sustituida por la CIE-11 a partir del 1 de enero del 2022, tras ser presentada en junio del 2018 y aprobada en mayo del 2019 en la Asamblea Mundial de la Salud. Sin embargo, su entrada en vigor se ve rodeada de cierta polémica.

Todos los sistemas de clasificación relacionados con la salud —y la CIE no es una excepción— se han ido adaptando a los conocimientos de cada momento histórico en que se han diseñado, no solo a los saberes médicos y científicos, sino también a las necesidades propias de los sistemas de salud, a los que finalmente sirven. Esta necesaria adaptación ha supuesto en muchos casos el abandono de algunos términos o categorías diagnósticas y la adopción de otras que definan mejor el concepto o armonicen de manera más precisa con el estado del arte, y la opinión de los expertos y la sociedad. Así, se han modificado y han llegado a desaparecer categorías asociadas con las conductas sexuales o se ha perfilado con mayor precisión la categorización de algunos trastornos, como las demencias. Ninguna herramienta es perfecta y la CIE-11, por primera vez, ha creado el mecanismo para adecuar dinámicamente la clasificación a medida que se detecte la necesidad de algún cambio a través de una plataforma de mantenimiento (2).

Los aspectos relacionados con el proceso de envejecimiento y su etapa final, la vejez, también se han visto afectados por estos cambios. Quizá el cambio más afortunado ha sido la eliminación de la categoría “senilidad”. Sin embargo, la clasificación sigue adoleciendo de deficiencias terminológicas que probablemente reflejan una escasa adaptación a los marcos teóricos y conceptuales vigentes en el campo del envejecimiento e, incluso, a estrategias propuestas y aprobadas por la propia OMS en aras de perfeccionar y mejorar la atención a la salud de la población mayor, varios de los cuales se discuten a continuación.

## LA CIE-11 Y LAS PERSONAS MAYORES

La estructura de las poblaciones y la situación socioeconómica de ellas determinan en gran medida su salud, sus principales enfermedades y sus causas de muerte. Sin embargo, no es sino hasta las últimas décadas que la transición demográfica —un fenómeno global, rápido y que se presenta acompañado de una transición epidemiológica— ha transformado el panorama sanitario y ejerce presión sobre la organización de los sistemas de salud en todo el mundo. Tanto la transición demográfica como la epidemiológica han cambiado el modo y el momento en que enfermamos y han llevado a modificar lo que entendemos por enfermedad y sus consecuencias. En la actualidad, algunos autores llaman a reconocer que nos enfrentamos a una tercera transición: la transición clínica (3) y de los sistemas de salud. Ante esta nueva transición se deben modificar los modos de entender, hacer y gestionar los cambios necesarios, y tomar en cuenta sus consecuencias en los campos de la clínica y la salud pública.

Durante estas transiciones hemos visto prolongarse de manera sustancial la duración de la vida, con un aumento en la esperanza de vida al nacer de más del doble en los últimos 100 años (4). Esto ha llevado a una mayor longevidad (5) y una vejez más larga por la expansión de la vida saludable y activa, y por los ajustes sociales y económicos que hacen los países, como la postergación de la edad de jubilación. En este contexto, es esencial y necesario transformar en toda la sociedad conceptos, mitos y estereotipos sobre el envejecimiento y la vejez, particularmente en el campo de la salud.

El envejecimiento, un proceso que se inicia al final del desarrollo de la persona, está influido por multitud de factores ambientales y genéticos. Algunos de ellos, incluso, pueden tener su origen en etapas tan tempranas como la programación fetal del desarrollo. Estos cambios, muchos de ellos deletéreos, condicionan cierta pérdida de la capacidad de adaptación. A lo largo de la vida, la suma de los cambios fisiológicos con la acción de ciertas noxas causales acaba generando consecuencias adversas a pesar de los procesos moduladores y adaptativos. Estos cambios conducen a la pérdida de algunas funciones y, finalmente, a la muerte. Si bien estas transformaciones se entremezclan con los procesos patológicos, según el conocimiento actual, definitivamente no pueden clasificarse como enfermedades (6).

En este contexto, la vejez no es sino la etapa final de la vida a la que conduce el proceso de envejecimiento y que inevitablemente lleva a la muerte. Bajo este marco conceptual, la vejez es el momento en el que se hacen evidentes los cambios fenotípicos resultantes tanto de la alostasis como del resto de los cambios sufridos a lo largo de la vida y de las modificaciones de la capacidad adaptativa que de ellos resultan (7). Esto también hace que la vejez sea muy heterogénea entre las personas y a todos los niveles de la biología.

Estos eventos son de una relevancia tal para la sociedad que han llevado a muchas organizaciones a replantearse el significado del envejecimiento e, incluso, su propia definición. Entre ellas se encuentra la propia OMS, que en su *Informe Mundial sobre el Envejecimiento y la Salud*, de 2015 (8), define el “envejecimiento saludable” como el proceso que permite al individuo mantener una vida funcional adaptada a sus propios intereses. Según esta definición, una persona de edad avanzada puede tener muchas enfermedades y tener un envejecimiento saludable si estas no modifican de manera suficiente su vida. Por primera vez se alejaban los conceptos de “saludable” y “enfermedad”, cambiando —con base en un enfoque de capacidades— el binomio salud-enfermedad por el de salud-deterioro funcional o salud-pérdida de autonomía funcional. En consecuencia, y en función de otras circunstancias concomitantes cuya consideración excede el propósito de este artículo, se propuso la modificación de los sistemas de salud y de los servicios de atención hacia un modelo fundamentado en los servicios integrales, coordinados y continuados (9).

A partir de estas consideraciones, se deberá revisar dónde y cómo se usa el término “vejez” (“*old age*”) en la CIE-11 —si es que debe utilizarse— y cómo se nombra la etapa de la vejez referida en ella como “períodos *geriátricos* inicial y final”, lo que implica una medicalización de esta etapa de la vida, al contrario de lo que se hace con otras que se llaman por su nombre, como la niñez, la adolescencia o la adultez.

Aunque en la vejez hay una mayor prevalencia de enfermedades y de discapacidades, equiparar esta etapa vital a las enfermedades y trastornos de salud que en ella puedan aparecer, aun cuando sean muy frecuentes, es una mixtificación, a nuestro modo de ver, equivocada. Más aun cuando para la mayoría de quienes alcanzan esa etapa de la vida, este sigue siendo un período de vida plena y, como lo define la propia OMS, saludable. Es cierto que la CIE no solo clasifica enfermedades, sino también los períodos de la vida y los “problemas relacionados con la salud”; sin embargo, no es menos cierto que la vejez por sí sola no representa un problema relacionado con la salud para muchos de quienes se encuentran en esta etapa de la vida.

Es importante encontrar términos más convenientes para referirnos a este período de la vida y a las personas que sobrevivieron a las etapas tempranas de la existencia y llegan a una vejez activa, de manera que invite a buscar oportunidades de mejorar la salud, la funcionalidad y la dignidad en esta etapa y esas personas.

Consideramos imprescindible cambiar, o al menos matizar, el epígrafe “vejez” de la CIE-11 para que no se perciba como un síntoma, signo o resultado clínico anómalo (como sugiere la clasificación), y se abra la opción de introducir otros términos no incluidos y que reflejan mucho mejor el estado de envejecimiento patológico. Entre los términos que gozan de un creciente soporte experimental y bibliográfico están, por ejemplo, “fragilidad” y “pérdida de la capacidad intrínseca”, que aportan mucha mayor precisión a la hora de definir la condición de la persona que no goza de un envejecimiento saludable (10).

La OMS debería abordar y resolver la contradicción interna que supone para una misma organización predicar y promover un “envejecimiento saludable” —para lo que ha logrado una concertación mundial— y al mismo tiempo colocar esta etapa de la vida en una clasificación de situaciones morbosas o de riesgo. Esta contradicción resulta particularmente difícil de justificar al inicio de la Década del Envejecimiento Saludable, que persigue establecer una plataforma para la innovación y el cambio (prioridad 1), recopilar mejores datos globales sobre el envejecimiento saludable (prioridad 3), alinear los sistemas de salud con las necesidades de las personas mayores (prioridad 5), sentar las bases para un sistema de atención a largo plazo en todos los países (prioridad 6) y emprender una campaña mundial para combatir la discriminación por edad (prioridad 8) (11).

En ese mismo tenor, es prioritario revisar y actualizar el modo de clasificar los padecimientos y trastornos asociados con el envejecimiento, empezando por el sistema de clasificación más importante en el mundo: la CIE-11.

## Declaración.

Las opiniones expresadas en este artículo son responsabilidad de los autores y no reflejan necesariamente los criterios ni la política de la *Revista Panamericana de Salud Pública / Pan American Journal of Public Health* y/o de la Organización Panamericana de la Salud.

## References

[1] 1. World Health Organization. History of the development of the ICD. Geneva: WHO [acceso el 5 de julio del 2021]. Disponible en: https://www.who.int/classifications/icd/en/HistoryOfICD.pdf

[2] 2. Organización Mundial de la Salud. Clasificación Internacional de Enfermedades, 11.^a^ revisión. Estandarización mundial de la información de diagnóstico en el ámbito de la salud. Ginebra: OMS; 2019 [acceso el 5 de julio del 2021]. Disponible en: https://icd.who.int/es

[3] 3. Rodríguez-Mañas L, Rodríguez-Artalejo F, Sinclair AJ. The third transition: The clinical evolution oriented to the contemporary older patient. J Am Med Dir Assoc. 2017;18:8−9. 10.1016/j.jamda.2016.10.005 27887892

[4] 4. Scitable by Nature Education. Life expectancy around the world has increased steadily for nearly 200 years [Internet] [acceso el 5 de julio del 2021]. Disponible en: https://www.nature.com/scitable/content/life-expectancy-around-the-world-has-increased-19786/

[5] 5. Gratton L, Scott A. The 100-year life: Living and working in an age of longevity. London: Bloomsbury Information Ltd; 2016.

[6] 6. Partridge L, Deelen J, Slagboom PE. Facing up to the global challenges of ageing. Nature. 2018;561:45−56. 10.1038/s41586-018-0457-8 30185958

[7] 7. Vineis P, Avendano-Pabon M, Barros H, Bartley M, Carmeli C, Carra L, et al. Special report: The biology of inequalities in health. Front Public Health. 2020;8:118 [acceso el 5 de julio del 2021]. Disponible en: 10.3389/fpubh.2020.00118 PMC7235337 32478023

[8] 8. World Health Organization. World report on ageing and health. Geneva: WHO; 2015 [acceso el 5 de julio del 2021]. Disponible en: https://apps.who.int/iris/handle/10665/186463

[9] 9. Beard JR, Officer A, de Carvalho IA, Sadana R, Pot AM, Michel JP, et al. The World Report on Ageing and Health: A policy framework for healthy ageing. Lancet. 2016;387:2145−54. 10.1016/S0140-6736(15)00516-4 PMC4848186 26520231

[10] 10. Belloni G, Cesari M. Frailty and intrinsic capacity: Two distinct but related constructs. Front Med. 2019;6:133 [acceso el 5 de julio del 2021]. Disponible en: 10.3389/fmed.2019.00133 PMC6591451 31275941

[11] 11. Organización Mundial de la Salud. Década del envejecimiento saludable 2021-2030. Ginebra: OMS; 2020 [acceso el 5 de julio del 2021] Disponible en: https:www.who.int/es/initiatives/decade-of-healthy-ageing

